# Associations between cognitive activities and all-cause mortality among older adults with cognitive impairment: A prospective cohort study

**DOI:** 10.1371/journal.pone.0319093

**Published:** 2025-02-20

**Authors:** Linjia Duan, Liming Zhao, Ziqiong Wang, Lu Liu, Ningying Song, Sen He

**Affiliations:** 1 Department of Cardiology, West China Hospital, Sichuan University, Chengdu, China; 2 West China School of Nursing, Sichuan University, Chengdu, China; 3 Department of Cardiology, Hospital of Chengdu Office of People’s Government of Tibetan Autonomous Region, Chengdu, China; 4 Department of Otolaryngology‑Head & Neck Surgery, West China Hospital, Sichuan University, Chengdu, China; 5 Department of Cardiology, Karamay Hospital of Integrated Chinese and Western Medicine, Karamay, China; Royal College of Surgeons in Ireland, IRELAND

## Abstract

**Background:**

Evidence on the association between cognitive activities and mortality among older adults with cognitive impairment is limited. Therefore, the study aimed to assess the association and examine whether baseline cognitive function mediates the association.

**Methods:**

A total of 10477 older participants with cognitive impairment (median age: 95.0 [IQR: 88.0–100.0], males: 27.9%, Mini-Mental State Examination score ≤24 points) from the Chinese Longitudinal Healthy Longevity Survey conducted between 1998 and 2014 were included, with follow-up until 2018. Exposures were three prevalent cognitive activities among older adults in China: reading books/newspapers, playing cards/mah-jong, and watching TV or listening to radio, and the outcome was all-cause mortality within a 10-year follow-up period. We evaluated the association between these activities and mortality using Cox regression models and also conducted a mediation analysis to examine the role of baseline cognitive function in this association.

**Results:**

During a follow-up period of totaling 33632.1 person-years, there were 8763 recorded deaths (83.6%). For each cognitive activity, the risk of mortality decreased with an increased frequency of engagement in these activities. Moreover, the risk of mortality significantly decreased with a greater number of cognitive activities. With zero activities as reference, adjusted hazard ratios were 0.83 (95% CI: 0.79–0.87) for one activity, 0.76 (95% CI: 0.69–0.83) for two activities, and 0.67 (95% CI: 0.53–0.86) for three activities, respectively. Stratified and sensitivity analyses confirmed the robustness of these findings. Additionally, baseline cognitive function partially mediated the association between cognitive activities and mortality; compared to zero activities, the mediated proportions were 15.2% (95% CI: 10.9%–22.4%) for one activity, 13.4% (95% CI: 8.9%–21.3%) for two activities, and 9.3% (95% CI: 4.2%–23.4%) for three activities, respectively.

**Conclusions:**

Among older adults with cognitive impairment in China, the risk of all-cause mortality significantly decreased as both the frequency and number of cognitive activities increased. Baseline cognitive function only mediated a small proportion of the benefits of cognitive activities in longevity.

## Introduction

Population aging represents one of the significant challenges confronting many countries worldwide, and cognitive impairment is notably prevalent among older adults, with its incidence increasing as age advances [1–3]. Moreover, numerous studies have shown that cognitive impairment is associated with an increased risk of death [4–6]. Inevitably, the increasing mortality rate among older adults with cognitive impairment will emerge as one of the major threats to future public health resources. On the other hand, cognitive activities have been a hot topic in the research field of older adults, and there is a large and growing body of evidence on the health benefits of engagement in cognitive activities [7–16]. Especially, emerging studies have presented that most cognitive activities potentially lead to a decreased risk of mortality [11,15,17–20].

Currently, important gaps between cognitive activities and mortality remain. First, previous studies about the association between cognitive activities and mortality mainly focused on the overall older adult population [11,15,17–20], without distinguishing those with abnormal cognitive levels from the general cohort. Second, cognitive function is commonly viewed as a mediator between cognitive activities and mortality [15,20]; furthermore, given the increased mortality risk with cognitive impairment [4–6], cognitive impairment might alleviate the benefits of cognitive activities in longevity. Therefore, evaluating the contribution of cognitive function is crucial for understanding the association between cognitive activities and mortality, particularly among older adults with cognitive impairment.

To address these knowledge gaps, the present study aimed to investigate the association between cognitive activities and all-cause mortality among older adults with cognitive impairment in China, using data from the Chinese Longitudinal Healthy Longevity Survey (CLHLS). In this study, we also investigated whether baseline cognitive function served as a mediator in the association between cognitive activities and all-cause mortality.

## Methods

### Study participants

The CLHLS is a nationwide, ongoing prospective cohort study focusing on community-dwelling older adults in China, and its general goal is to enhance our understanding of the determinants of healthy aging. Detailed information has been reported elsewhere [11,21]. Briefly, the baseline survey of the CLHLS commenced in 1998, with follow-up surveys conducted every two to three years. Data from eight surveys have been made available, specifically for the years 1998, 2000, 2002, 2005, 2008, 2011, 2014, and 2018. In the initial year of the study (1998), a multistage stratified cluster sampling method was employed to conduct the baseline survey across a randomly selected half of the counties and cities within 22 out of China’s 31 provinces, and the population residing in these surveyed areas represented approximately 85.0% of the total Chinese population. To mitigate attrition due to mortality and loss to follow-up, new participants have been enrolled since wave 2000, with finally covering 23 of 31 provinces in China. The surveys were conducted in the homes of participants by trained interviewers utilizing a structured questionnaire. In instances where participants were unable to respond, proxy respondents—typically a spouse or other close family members—were interviewed; however, questions pertaining to cognitive function and mood were answered directly by the participants themselves. The study was carried out in accordance with the principles outlined in the Declaration of Helsinki and received approval from the Research Ethics Committee of Peking University (IRB00001052–13074). All participants, or their proxy respondents, provided written informed consent.

**Fig 1:**
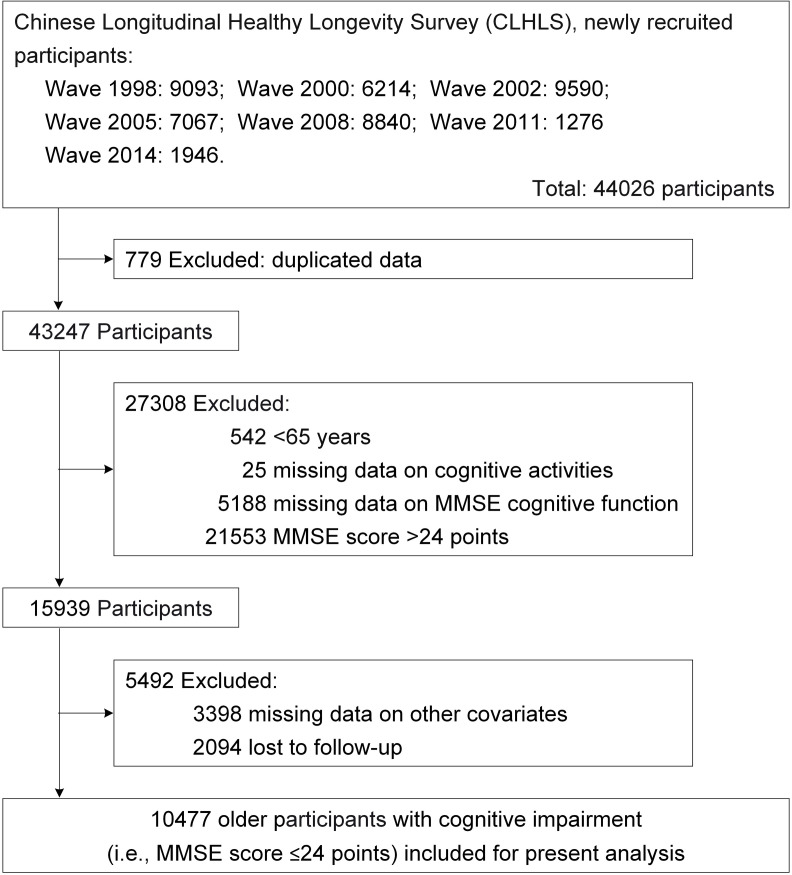
Flow chart of participants selection. MMSE=mini-mental state examination.

The present study was based on seven waves (i.e., 1998, 2000, 2002, 2005, 2008, 2011, and 2014) within the CLHLS, and the final wave of interview was 2018. In the CLHLS study, cognitive function was assessed using the Chinese version of the Mini-Mental State Examination (MMSE) [6], which evaluates various domains of cognitive functioning, including calculation, language, orientation, and recall. The Chinese version of the MMSE takes into account the cultural and socioeconomic contexts specific to China, ensuring that all items in the test are understood and answered by participants with normal cognitive abilities. The validity and reliability of the Chinese version of the MMSE have been established by previous studies [21,22]. In the study, we classified responses of “unable to answer” as “incorrect” in the MMSE based on the literatures [23,24], and the total MMSE score ranged from 0 to 30 points, with higher scores indicating better cognitive function. Cognitive impairment was defined as a total MMSE score of ≤ 24 points [6,12]. A flow chart illustrating the participant enrollment process for this study is presented in Fig 1, and the final sample comprised 10477 older participants (aged ≥ 65 years) with cognitive impairment.

### Assessment of cognitive activities

Participants were interviewed about their engagement in three most popular cognitive activities among older adults in China, and these activities included reading books/newspapers, playing cards/mah-jong and watching TV or listening to radio. Participants reported the frequency of their participation using categories such as “never,” “sometimes,” or “almost everyday” for the waves conducted in 1998 and 2000. For subsequent waves in 2002, 2005, 2008, 2011, and 2014, participants used a more detailed scale: “never,” “not monthly but sometimes,” “not weekly but at least once a month,” “not daily but once a week,” or “almost everyday.” We recoded these responses to create a uniform scale across the seven waves: (1) never: never; (2) sometimes: sometimes, not monthly/but sometimes, not weekly/but at least once for a month, not daily/but once for a week; (3) almost everyday: almost everyday.

To explore the effect of cumulative participation in cognitive activities on mortality, we then constructed a dichotomous variable for participation in cognitive activities by assigning a value of 0 for the scale (i.e., never) and 1 for the scales (i.e., sometimes and almost everyday), and we further summed the three dichotomised variables to create a cumulative measure, resulting in a cumulative cognitive activity variable with values ranging from zero to three.

### Study outcome

The outcome of the study was all-cause mortality within a 10-year follow-up period. Information regarding survival status and date of death was collected through interviews with close family members during each survey and corroborated using death certificates, hospital admission records, and medical records when available. All participants were followed from the initial interview until either the occurrence of the outcome or their most recent interview.

### Assessment of covariates

In the study, we also investigated a comprehensive range of factors that have been associated with cognitive activities and mortality. These factors include: sex, age, education level, marital status, place of residence, co-residence, regular consumption of fruits, regular consumption of vegetables, regular intake of meats, current smoking habits, patterns of alcohol consumption, exercise routines, and history of comorbidities (hypertension, diabetes, heart diseases, cerebrovascular diseases, respiratory diseases, and cancer), as well as self-rated health assessments. Detailed information regarding the scales utilized for these factors in the present study can be found in S1 Table.

### Statistical analysis

S2 Table presents the distributions of baseline covariates with missing data. In the primary analysis, cases with missing data were excluded under the assumption of missing completely at random. Overall, the analyses consisted of four steps: (1) comparing baseline characteristics; (2) determinating the association between cognitive activities and all-cause mortality; (3) conducting stratified and sensitivity analyses; (4) assessing whether baseline cognitive function served as a mediator between cognitive activities and mortality.

Firstly, baseline characteristics were presented as median (interquartile range, IQR) for continuous variables and as number (percentage) for categorical variables. For continuous variables, the p-value for trend was calculated using the Pearson test when the row variable followed a normal distribution, and the Spearman test when it exhibited a non-normal distribution. In cases where the row variable was categorical, the p-value for trend was derived from the Mantel–Haenszel test of trend.

Secondly, the Kaplan-Meier method was used to estimate the cumulative incidence of all-cause mortality across various subgroups, with the log-rank test used for comparisons. Then, Cox proportional hazards models were used to estimate the association between cognitive activities and mortality, with follow-up time as the time scale, and we found no evidence indicating a potential violation of the proportional-hazards assumption regarding the exposure-outcome association (S1 Fig). We estimated the association between cognitive activities and mortality using two models: (1) model 1 included sex and age; and (2) model 2 incorporated sex, age, education, marital status, residence, co-residence, regular intake of fruits, regular intake of vegetables, regular intake of meats, current smoking, current drinking, current regular exercise, hypertension, diabetes, heart diseases, cerebrovascular diseases, respiratory diseases, cancer, and self-rated health.

Thirdly, to test the robustness of our primary findings, we repeated all analyses in various subgroups, along with several sensitivity analyses: (1) to address loss to follow-up, we conducted a sensitivity analysis by censoring losses at the end of follow-up time; (2) to exclude deaths that occurred within the first year or two years of follow-up, thereby reducing potential reverse causation; (3) to account for variations due to different waves, we adjusted for baseline interview timing; (4) to mitigate possible bias resulting from missing data, we implemented multiple imputation techniques in our analysis; and (5) to examine the possibility of unmeasured confounding between cognitive activities and mortality, we calculated E-values [25].

Finally, we performed a regression-based causal mediation analysis within the direct counterfactual framework to evaluate how baseline cognitive function, as measured by MMSE, mediate the association between cognitive activities and mortality, using the CMAverse package [26–28]. In brief, two regression models were fitted: one for the outcome (i.e., all-cause mortality; using a Cox proportional-hazards model) and another for the mediator (i.e., baseline cognitive function; using linear regression). In the two models, cognitive activities were treated as categorical variables, while the baseline cognitive function served as a linear term. Standard errors were estimated via bootstrapping with 1000 samples. All models were adjusted for potential confounders.

All analyses were conducted using R version 4.2.2 (available at http://www.R-project.org) with the implementation of essential packages, including “compareGroups”, “survival”, “mice”, “CMAverse”, and “stats”. All statistical tests employed were two-sided, and p-values less than 0.05 were considered statistically significant. In addition, the official organization (i.e., Center for Healthy Aging and Development, Peking University) of the CLHLS suggests that weights may not be necessary if researchers are only describing the sample’s status without comparing it to other populations. These suggestions are available on their website at http://chads.nsd.pku.edu.cn/yjxm/zglnjkyxysgztc/index.htm. Therefore, we did not incorporate the complex sampling design into our analyses.

## Results

### Baseline characteristics

The study comprised a total of 10477 older participants with cognitive impairment (median age: 95.0 [IQR: 88.0–100.0]; males: 27.9%). Table 1 presents the baseline characteristics of participants categorized by the number of cognitive activities. Participants engaging in a greater number of cognitive activities were more likely to be male, younger, better educated, married, and residing in urban areas. Additionally, there was a higher prevalence of comorbidities among those participating in more cognitive activities, and their MMSE scores were also elevated compared to those with fewer cognitive activities. Further detailed information regarding baseline characteristics is provided in Table 1. Moreover, baseline characteristics stratified by the frequency of cognitive activities are presented in S3–S5 Tables.

**Table 1:** Baseline characteristics by number of cognitive activities.

	All	Zero activities	One activity	Two activities	Three activities	p for trend
No. of participants	10447	5264	4246	831	106	
Sex: male	2916 (27.9%)	1173 (22.3%)	1212 (28.5%)	463 (55.7%)	68 (64.2%)	<0.001
Age (years)	95.0 (88.0–100.0)	97.0 (90.0–101.0)	93.0 (86.0–100.0)	91.0 (83.0–98.0)	88.0 (80.0–95.8)	<0.001
Education						<0.001
No school	8592 (82.2%)	4643 (88.2%)	3528 (83.1%)	393 (47.3%)	28 (26.4%)	
1 year or more	1855 (17.8%)	621 (11.8%)	718 (16.9%)	438 (52.7%)	78 (73.6%)	
Marital status						<0.001
Not in marriage	9085 (87.0%)	4807 (91.3%)	3575 (84.2%)	631 (75.9%)	72 (67.9%)	
In marriage	1362 (13.0%)	457 (8.7%)	671 (15.8%)	200 (24.1%)	34 (32.1%)	
Residence						<0.001
Urban	3698 (35.4%)	1460 (27.7%)	1726 (40.7%)	442 (53.2%)	70 (66.0%)	
Rural	6749 (64.6%)	3804 (72.3%)	2520 (59.3%)	389 (46.8%)	36 (34.0%)	
Co-residence						0.592
With family members	8711 (83.4%)	4312 (81.9%)	3620 (85.3%)	691 (83.2%)	88 (83.0%)	
Alone	1351 (12.9%)	791 (15.0%)	454 (10.7%)	98 (11.8%)	8 (7.5%)	
In an institution	385 (3.7%)	161 (3.1%)	172 (4.1%)	42 (5.1%)	10 (9.4%)	
Regular intake of fruits	2415 (23.1%)	912 (17.3%)	1160 (27.3%)	302 (36.3%)	41 (38.7%)	<0.001
Regular intake of vegetables	8179 (78.3%)	3911 (74.3%)	3487 (82.1%)	695 (83.6%)	86 (81.1%)	<0.001
Regular intake of meats	3827 (36.6%)	1659 (31.5%)	1747 (41.1%)	371 (44.6%)	50 (47.2%)	<0.001
Current smoking	1369 (13.1%)	541 (10.3%)	618 (14.6%)	176 (21.2%)	34 (32.1%)	<0.001
Current drinking	1893 (18.1%)	878 (16.7%)	801 (18.9%)	185 (22.3%)	29 (27.4%)	<0.001
Current regular exercise	1980 (19.0%)	612 (11.6%)	1016 (23.9%)	301 (36.2%)	51 (48.1%)	<0.001
Hypertension	1459 (14.0%)	669 (12.7%)	635 (15.0%)	138 (16.6%)	17 (16.0%)	<0.001
Diabetes	110 (1.1%)	45 (0.9%)	45 (1.1%)	15 (1.8%)	5 (4.7%)	0.001
Heart diseases	650 (6.2%)	255 (4.8%)	315 (7.4%)	74 (8.9%)	6 (5.7%)	<0.001
Cerebrovascular diseases	393 (3.8%)	182 (3.5%)	159 (3.7%)	47 (5.7%)	5 (4.7%)	0.010
Respiratory diseases	1147 (11.0%)	544 (10.3%)	476 (11.2%)	118 (14.2%)	9 (8.5%)	0.011
Cancer	30 (0.3%)	16 (0.3%)	9 (0.2%)	4 (0.5%)	1 (0.9%)	0.568
Self-rated health						<0.001
Poor	1678 (16.1%)	984 (18.7%)	586 (13.8%)	94 (11.3%)	14 (13.2%)	
Fair	3774 (36.1%)	2010 (38.2%)	1469 (34.6%)	261 (31.4%)	34 (32.1%)	
Good	4995 (47.8%)	2270 (43.1%)	2191 (51.6%)	476 (57.3%)	58 (54.7%)	
Reading books/newspapers						<0.001
Never	9854 (94.3%)	5264 (100.0%)	4169 (98.2%)	421 (50.7%)	0 (0.0%)	
Sometimes	341 (3.3%)	0 (0.0%)	47 (1.1%)	224 (27.0%)	70 (66.0%)	
Almost everyday	252 (2.4%)	0 (0.0%)	30 (0.7%)	186 (22.4%)	36 (34.0%)	
Playing cards/mah-jong						<0.001
Never	9780 (93.6%)	5264 (100.0%)	4116 (96.9%)	400 (48.1%)	0 (0.0%)	
Sometimes	478 (4.6%)	0 (0.0%)	93 (2.2%)	310 (37.3%)	75 (70.8%)	
Almost everyday	189 (1.8%)	0 (0.0%)	37 (0.9%)	121 (14.6%)	31 (29.2%)	
Watching TV or listening to radio						<0.001
Never	5481 (52.5%)	5264 (100.0%)	207 (4.9%)	10 (1.2%)	0 (0.0%)	
Sometimes	2653 (25.4%)	0 (0.0%)	2284 (53.8%)	336 (40.4%)	33 (31.1%)	
Almost everyday	2313 (22.1%)	0 (0.0%)	1755 (41.3%)	485 (58.4%)	73 (68.9%)	
MMSE score	19.0 (14.0–22.0)	17.0 (11.0–21.0)	20.0 (15.0–23.0)	21.0 (18.0–23.0)	22.0 (20.0–23.0)	<0.001

Values are median (IQR) or n (%). For continuous variables, the p-value for trend was calculated using the Pearson test for normal distributions and the Spearman test for non-normal distributions. For categorical variables, it was derived from the Mantel–Haenszel test.

Abbreviations: IQR, interquartile range; MMSE, mini-mental state examination.

### Associations between cognitive activities and all-cause mortality

During a follow-up period of 33632.1 person-years, a total of 8763 deaths (83.6%) were recorded. For the three individual cognitive activities, Kaplan-Meier curves indicated that a higher frequency of cognitive activities was associated with a more favorable clinical outcome (all log-rank p < 0.001, S2 Fig). Both univariate and multivariate analyses further demonstrated that an increased frequency of cognitive activities was associated with lower mortality risk (S6 Table).

**Fig 2:**
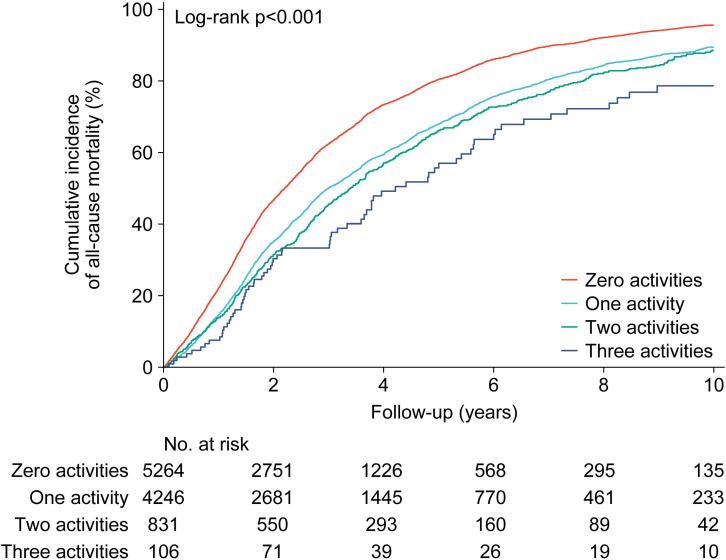
Kaplan-Meier analysis showing cumulative incidence of all-cause mortality by number of cognitive activities.

Furthermore, we assessed the association between the number of cognitive activities and all-cause mortality. As the number of cognitive activities increased, there was a significant decrease in mortality rates (log-rank p < 0.001, Fig 2). Accordingly, when comparing participants without any cognitive activity (zero activities), crude mortality risks were lower for those engaging in one activity (hazard ratios [HR] = 0.70, 95% CI: 0.67–0.73), two activities (HR = 0.64, 95% CI: 0.59–0.70), and three activities (HR = 0.50, 95% CI: 0.39–0.63), respectively (Table 2). When using zero activities as the reference, adjusted HRs were 0.83 for one activity (95% CI: 0.79–0.87), 0.76 for two activities (95% CI: 0.69–0.83), and 0.67 for three activities (95% CI: 0.53–0.86), respectively (Table 2).

**Table 2:** Associations of number of cognitive activities with all-cause mortality and medication analysis of number of cognitive activities by baseline cognitive function.

	Zero activities	One activity	Two activities	Three activities
Number of participants	5264	4246	831	106
Number of deaths	4675	3389	630	69
Total person-years of follow-up	14904.5	15220.4	3072.4	434.7
Mortality rate (95% CI), per 1000 person-years	313.7 (306.2–321.1)	222.7 (216.1–229.3)	205.0 (190.8–219.3)	158.7 (124.4–193.1)
Unadjusted HR (95% CI), p	1.00 (ref)	0.70 (0.67–0.73), < 0.001	0.64 (0.59–0.70), < 0.001	0.50 (0.39–0.63), < 0.001
Adjusted HR (95% CI), p				
model 1^a^	1.00 (ref)	0.83 (0.79–0.86), < 0.001	0.77 (0.70–0.84), < 0.001	0.70 (0.55–0.89), 0.003
model 2^b^	1.00 (ref)	0.83 (0.79–0.87), < 0.001	0.76 (0.69–0.83), < 0.001	0.67 (0.53–0.86), 0.001
*Sensitivity analysis*_*HR (95% CI), p*_				
Losses censored at the end of follow-up^c^	1.00 (ref)	0.86 (0.82–0.90), < 0.001	0.81 (0.74–0.88), < 0.001	0.73 (0.58–0.94), 0.013
Excluding deaths within the first year^c^	1.00 (ref)	0.87 (0.82–0.91), < 0.001	0.80 (0.72–0.88), < 0.001	0.78 (0.60–1.01), 0.056
Excluding deaths within the first two years^c^	1.00 (ref)	0.86 (0.81–0.92), < 0.001	0.81 (0.72–0.92), < 0.001	0.65 (0.47–0.91), 0.011
Adjustment for the waves^d^	1.00 (ref)	0.85 (0.81–0.89), < 0.001	0.78 (0.71–0.85), < 0.001	0.67 (0.53–0.86), 0.001
After multiple imputation^e^	1.00 (ref)	0.81 (0.78–0.85), < 0.001	0.74 (0.68–0.80), < 0.001	0.66 (0.53–0.82), < 0.001
E-values^f^		1.69	1.97	2.34
*Mediation analysis* ^g^				
Natural direct effect; HR (95% CI), p		0.86 (0.82–0.90), < 0.001	0.79 (0.73–0.85), < 0.001	0.70 (0.57–0.88), 0.004
Natural indirect effect; HR (95% CI), p		0.97 (0.96–0.98), < 0.001	0.96 (0.95–0.97), < 0.001	0.96 (0.94–0.98), < 0.001
Mediation proportion; % (95% CI), p		15.2 (10.9–22.4), < 0.001	13.4 (8.9–21.3), < 0.001	9.3 (4.2–23.4), 0.002

^a^Adjustment for sex and age.

^b^Adjustment for sex, age, education, marital status, residence, co-residence, regular intake of fruits, regular intake of vegetables, regular intake of meats, current smoking, current drinking, current regular exercise, hypertension, diabetes, heart diseases, cerebrovascular diseases, respiratory diseases, cancer, and self-rated health.

^c^Adjustment for the covariates as in model 2.

^d^Adjustment for the covariates as in model 2 plus the waves.

^e^Multiple imputation was performed by chained equations to create 5 datasets, of which the resultant model estimates for each were combined using Rubin`s rules. The present sample size was a little different from the sample size in the flow chart, which was caused by the partial overlap between the lost participants and the participants with missing baseline data.

^f^E-values were for the adjusted HRs as shown in model 2.

^g^Natural direct effect and natural indirect effect estimated the effect of number of cognitive activities on all-cause mortality that did not or did act through the mediator (i.e., baseline cognitive function), respectively. The mediation proportion estimated the percentage of the effect of number of cognitive activities, on the log(HR) scale, that acted through the mediator (i.e., baseline cognitive function). The model was adjusted for sex, age, education, marital status, residence, co-residence, regular intake of fruits, regular intake of vegetables, regular intake of meats, current smoking, current drinking, current regular exercise, hypertension, diabetes, heart diseases, cerebrovascular diseases, respiratory diseases, cancer, and self-rated health.

Abbreviations: CI, confidence interval; HR, hazard ratio.

### Stratified and sensitivity analyses

**Fig 3:**
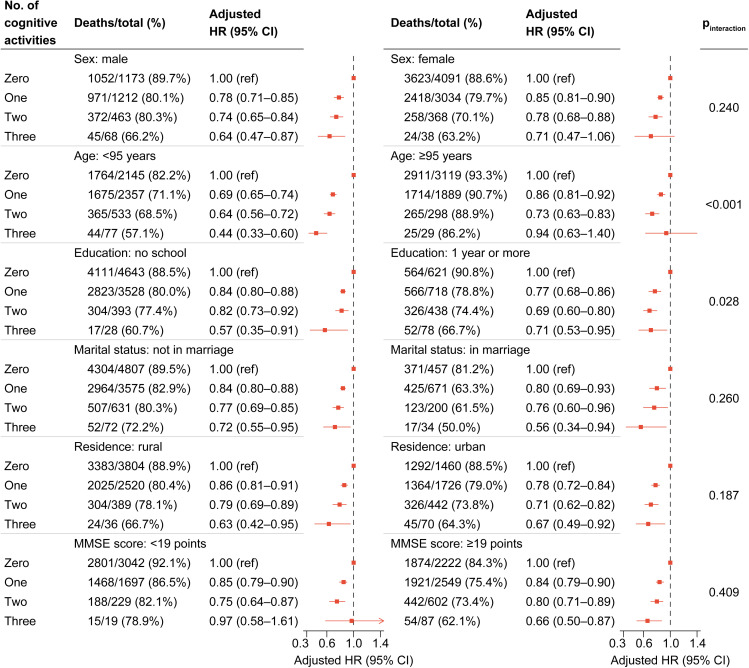
Stratified analyses by potential modifiers of the association between the number of cognitive activities and all-cause mortality.

We further performed stratified analyses to assess the association between the number of cognitive activities (zero activities vs. one activity vs. two activities vs. three activities) and the risk of all-cause mortality across various subgroups. Overall, we observed a decrease in mortality risk with an increasing number of cognitive activities in all subgroups (Fig 3, S3 Fig). None of the variables—including sex, age, education, marital status, residence, MMSE score, current smoking, current drinking, hypertension, and cerebrovascular diseases—significantly modified the association between the number of cognitive activities and mortality. Moreover, the sensitivity analyses did not find substantial changes in the results (Table 2).

Each stratification adjusted for all factors (sex, age, education, marital status, residence, co-residence, regular intake of fruits, regular intake of vegetables, regular intake of meats, current smoking, current drinking, current regular exercise, hypertension, diabetes, heart diseases, cerebrovascular diseases, respiratory diseases, cancer, and self-rated health) except the stratification factor itself. The grouping criteria for continuous variables were determined based on the median values. Due to the extensive size of the figure, certain information is included in S3 Fig.

### Mediation analysis

Table 2 also presents the results of mediation analysis that assessed whether baseline cognitive function served as a mediator between the number of cognitive activities and all-cause mortality. The results indicated that both the natural direct effect and the natural indirect effect were statistically significant, suggesting a partial mediation effect of baseline cognitive function on the association between the number of cognitive activities and mortality. Specifically, when comparing with zero activities, the proportion mediated by baseline cognitive function was found to be 15.2% (95% CI: 10.9%–22.4%) for one activity, 13.4% (95% CI: 8.9%–21.3%) for two activities, and 9.3% (95% CI: 4.2%–23.4%) for three activities, respectively. Furthermore, we also evaluated whether baseline cognitive function acted as a mediator between individual cognitive activities and mortality; these results are detailed in S7 Table.

Additionally, another mediation analysis was performed to examine the directionality of effects by using baseline cognitive function as the predictor variable and cognitive activities as the mediator variable; however, all natural indirect effects were found to be non-significant (S8 Table).

## Discussion

In analyses based on a large cohort of older adults with cognitive impairment, we found that frequent participation in cognitive activities—such as reading books/newspapers, playing cards/mah-jong, and watching TV or listening to radio—was associated with a lower risk of all-cause mortality. Furthermore, the risk of mortality also significantly decreased with an increased number of cognitive activities. Moreover, baseline cognitive function only mediated a small proportion of the benefits of cognitive activities in longevity.

Engagement in cognitive activities is associated with reduced mortality risk. Emerging evidence indicates reading books/newspapers may have a beneficial impact on survival [11,20,29]. Similarly, our findings are consonant with these studies, and the underlying mechanisms might be partially attributed to the inverse association of reading books/newspapers with cognitive impairment [20,30,31]. In contrast, some previous found reading had no effect on mortality [15,32], and the inconsistency might be the caused by the differences of study populations. In accordance with our results, some previous studies also showed playing cards/mah-jong reduced risk of all-cause mortality among older adults [11,15,18]. Playing cards/mah-jong is inherently social in nature, which has be associated with a reduced mortality risk [33]. For watching TV or listening to radio, some previous studies have suggested that watching TV for a long time might be associated with increased mortality risk [34,35]; on the other hand, a study reported positive effect of more participation in watching TV and listening to radio on decreased mortality risk among oldest-old adults [11]. Combined with these studies [11,34,35], the present findings might suggest increased frequency of watching TV may be associated with a decreased mortality risk, but excessively prolonged viewing time may increase mortality risk. Meanwhile, most of present study participants were illiterate and thus lacked many sources of information; therefore, watching TV or listening to the radio may maintain cognitive function, further reducing mortality risk [17]. Additionally, most studies have shown cognitive activities are also associated with other factors related to better health status. For example, cognitive activities are associated with a lower risk of activities of daily living disability [10], and those who participate more in cognitive activities may have a better consciousness of diseases and they may access the medical care system more actively [36]. Despite the diverse characteristics of the samples in these studies, a consistent positive association has been observed between cognitive activities and health. Furthermore, the present study indicated the risk of all-cause mortality progressively decreased with increased number of cognitive activities. Similarly, some previous studies [15,37], also displayed older adults were less possibly to die among those engaged in an increased number of activities than those who engaged in few activities. Although the p-values for interactions concerning age and education were less than 0.050 in the study, these findings may lack significant clinical implications due to multiple testing considerations and similar directional trends in associations. Thus, both previous studies and the current investigation suggest that frequent engagement in cognitive activities, as well as participation in a greater variety of such activities, may reduce the risk of mortality. Our findings further extend these results to older adults with cognitive impairment.

The mediation analysis showed that only up to 27.9% of the association between cognitive activities and all-cause mortality was explained by baseline cognitive function, indicating that cognitive activities reducing mortality risk only depended on baseline cognitive function partly; in other words, even if the older adults had cognitive impairment, they may still get significant survival advantage by cognitive activities. Although the present findings showed baseline cognitive function only mediated a small proportion of the benefits of cognitive activities in mortality, another study [20], using the data from the Health and Retirement Study, suggested subsequent cognition was a complete mediator of the baseline book reading survival advantage after adjustment for baseline cognition in a total of 3635 older participants. Whether subsequent cognition was a complete mediator between cognitive activities and all-cause mortality in the older adults with cognitive impairment, further studies are needed and warranted. We also found that cognitive activities did not mediate the association between baseline cognitive function and survival, indicating that reverse causality mediation was not present, and that is higher baseline cognitive level could not cause increased cognitive activities, further reducing mortality risk.

As far as we know, this is the largest prospective cohort study to assess the association of cognitive activities with the risk of all-cause mortality among older adults with cognitive impairment. The main strengths of this study include the community-based design, the large sample, and the control as many potential confounding factors as possible, as well as the robust findings of sensitivity analyses. Nevertheless, our study still had some limitations. First, the current sample consisted of older Chinese adults, which might limit the applicability of the results to other populations. Furthermore, a majority of participants lacked formal education and resided in rural areas, potentially constraining the generalizability of the findings. Nevertheless, results from stratified analyses revealed similar patterns among participants who had received formal education and lived in urban settings. Second, because the study was a secondary analysis of data, we only investigated three kinds of cognitive activities, which did not represent the full scale of cognitive activities among older adults in China. Future study considering comprehensive cognitive activities needs to be performed to confirm the positive association between cognitive activities and reduced risk of mortality. Third, some potential factors, either unmeasured or unknown, might confound the association between cognitive activities and mortality. While, E-value analysis could suggest the robustness of the findings in some degree. Fourth, the factors assessed did not include information about the duration, possibly leading to imprecise measurements. Fifth, ‘reverse causality’ might interpretate the longitudinal association; however, we did not find substantial changes after excluding deaths within the first year or the first two years. Finally, since the activities of reading books/newspapers, as well as playing cards/mah-jong, were infrequently engaged in, these activities might play a minor role in the sample.

## Conclusion

In conclusion, the study suggested that a greater frequency of participation in cognitive activities potentially resulted in lower risk of all-cause mortality among older adults with cognitive impairment. Furthermore, with an increased number of cognitive activities, mortality risk significantly decreased. Baseline cognitive function only mediated a small proportion of the benefits of cognitive activities in longevity among older adults with cognitive impairment; therefore, even if older adults experience cognitive decline, it is essential that they continue to engage positively in cognitive activities. Our findings may carry significant implications for public health policy and underscore the necessity of incorporating a diverse range of cognitive activities into the daily routines of older adults with cognitive impairment.

## Supporting information

S1 Table Definitions of baseline covariates in the present study. (PDF)

S2 Table Distributions of baseline covariates with missing data. (PDF)

S3 Table Baseline characteristics by reading books/newspapers. (PDF)

S4 Table Baseline characteristics by playing cards/mah-jong. (PDF)

S5 Table Baseline characteristics by watching TV or listening to radio. (PDF)

S6 Table Associations of individual cognitive activities with all-cause mortality. (PDF)

S7 Table Mediation analysis of individual cognitive activities by baseline cognitive function. (PDF)

S8 Table Mediation analysis of baseline cognitive function by cognitive activities. (PDF)

S1 Fig Testing proportional-hazards assumptions. Note: The proportional-hazards assumption is an important premise for Cox proportional-hazards models; however, no hazards are perfectly proportional in nearly any clinical study _(JAMA 2020;323:1401–1402)_. In small to moderate-sized samples, statistical tests may fail to reject such model assumptions due to insufficient power; however, even minor violations of the assumption can become evident with a sufficiently large sample size. Indeed, even when the model only approximately satisfies the proportional-hazards assumption, it can still yield reasonably accurate inferences _(Am J Epidemiol 2024;193:926–927)_. With this consideration in mind, it’s best to use a combination of statistical tests and visual tests to determine the most serious violations. For statistical tests, we assessed the proportional-hazards assumption using the `cox.zph` function from the `survival` package, applying a p-value threshold of 0.001 _(__①__Am J Epidemiol 2024;193(6):926–927;__②__Nat Med 2024;30:85–97)_. For visual tests, we visually assessed this assumption by examining the relationship between β(t) of exposures and the timescale. We found no evidence indicating a potential violation of the proportional-hazards assumption for the exposure-outcome association, with a p-value from the Schoenfeld individual tests exceeding 0.001. In addition, the relationship between β(t) of exposures and the timescale could be characterized as approximately horizontal and constant. Overall, both statistical analyses and graphical representations supported that the proportional-hazards assumption was satisfied, or at least approximately met, allowing for data analysis using Cox proportional-hazards models.
(PDF)

S2 Fig Kaplan-Meier analysis showing cumulative incidence of all-cause mortality by the frequency of individual cognitive activities. (PDF)

S3 Fig Stratified analyses by potential modifiers of the association between number of cognitive activities and all-cause mortality. Note: Each stratification with adjustment for all factors (sex, age, education, marital status, residence, co-residence, regular intake of fruits, regular intake of vegetables, regular intake of meats, current smoking, current drinking, current regular exercise, hypertension, diabetes, heart diseases, cerebrovascular diseases, respiratory diseases, cancer, and self-rated health) except the stratification factor itself.
(PDF)
