# Supplementary material for: Associations between cognitive activities and all-cause mortality among older adults with cognitive impairment: A prospective cohort study
Source: PLoS One. 2025 Feb 20;20(2):e0319093. doi: 10.1371/journal.pone.0319093 (PMC11841911; doi:10.1371/journal.pone.0319093)
Supplement: S1 Table — (PDF) [file pone.0319093.s001.pdf]

S1 Table. Definitions of baseline covariates in the present study

	Questions in the CLHLS questionnaire	Options for the questions	Scales of reclassification in the present study
Sex		· male<br>· female	· Male: male<br>· Female: female
Age			· Continuous (years)
Education	How many years did you attend school?	· years of school<br>· don't know<br>· missing	· No school: years of school =0<br>· 1 year or more: years of school ≥1<br>· missing: don't know, missing
Marital status	Current marital status?	· currently married and living with spouse<br>· separated<br>· divorced<br>· widowed<br>· never married<br>· don't know<br>· missing	· In marriage:<br>currently married and living with spouse<br>· Not in marriage:<br>separated, divorced, widowed, never married<br>· missing: don't know, missing
Residence	Current residence area of interviewee?	Wave 1998<br>· urban (city and town)<br>· rural<br><br>Waves 2000, 2002, 2005, 2008, 2011, 2014<br>· city<br>· town<br>· rural	· Urban: city, town<br>· Rural: rural
Co-residence	Co-residence?	· with family member(s)<br>· alone<br>· in a nursing home<br>· missing	· With family members: with household member(s)<br>· Alone: alone<br>· In an institution: in a nursing home<br>· missing: missing
Regular intake of fruits and vegetables, respectively	Do you eat these foods, respectively?	· almost everyday<br>· except winter/quite often<br>· occasionally<br>· rarely or never<br>· don't know<br>· missing	· Regular intake:<br>almost everyday, except winter/quite often<br>· No regular intake:<br>occasionally, rarely or never<br>· missing: don't know, missing
Regular intake of meats	Do you eat these foods, respectively?	Waves 1998, 2000, 2002, 2005<br>· almost everyday<br>· occasionally<br>· rarely or never<br>· missing<br><br>Waves 2008, 2011, 2014<br>· almost everyday	· Regular intake:<br>almost everyday; not everyday, but at least once per week<br>· No regular intake:<br>not every week, but at least once per month; not every month, but occasionally; occasionally; rarely or never.<br>· missing: don't know, missing

		· not everyday, but at least once per week<br>· not every week, but at least once per month<br>· not every month, but occasionally<br>· rarely or never<br>· don't know<br>· missing	
Current smoking	Do you smoke at present?	· yes<br>· no<br>· missing	· Current smoking: yes<br>· No smoking at present: no<br>· missing
Current drinking	Do you drink at present?	· yes<br>· no<br>· don't know<br>· missing	· Current drinking: yes<br>· No drinking at present: no<br>· missing: don't know, missing
Current regular exercise	Do you do exercises regularly at present?	· yes<br>· no<br>· don't know<br>· missing	· Current regular exercise: yes<br>· No regular exercise at present: no<br>· missing: don't know, missing
Hypertension, diabetes, heart diseases, cerebrovascular diseases, respiratory diseases, and cancer, respectively	Are you suffering from these diseases, respectively?	· yes<br>· no<br>· don't know<br>· missing	· Yes: yes<br>· No: no<br>· missing: don't know, missing
Self-rated health	How do you rate your health at present?	· very good<br>· good<br>· so so<br>· bad<br>· very bad<br>· not able to answer<br>· missing	· Good: very good, good<br>· Fair: so so<br>· Poor: bad, very bad<br>· missing: not able to answer, missing
MMSE score			· Continuous (points; treating responses of “unable to answer” as “wrong”)

Note:

More detailed information about these covariates can be found on: https://agingcenter.duke.edu/CLHLS.

Abbreviations: CLHLS = Chinese Longitudinal Healthy Longevity Surveys, MMSE=mini-mental state examination.
