# Supplementary material for: Associations between cognitive activities and all-cause mortality among older adults with cognitive impairment: A prospective cohort study
Source: PLoS One. 2025 Feb 20;20(2):e0319093. doi: 10.1371/journal.pone.0319093 (PMC11841911; doi:10.1371/journal.pone.0319093)
Supplement: S2 Table — (PDF) [file pone.0319093.s002.pdf]

S2 Table. Distributions of baseline covariates with missing data

	Number of missing data	Percentage of missing data (%)
Sex	0	0.0
Age	0	0.0
Education	142	0.9
Marital status	3	0.0
Residence	0	0.0
Co-residence	7	0.0
Regular intake of fruits	6	0.0
Regular intake of vegetables	8	0.1
Regular intake of meats	29	0.2
Current smoking	4	0.0
Current drinking	8	0.1
Current regular exercise	12	0.1
Hypertension	943	5.9
Diabetes	973	6.1
Heart diseases	906	5.7
Cerebrovascular diseases	879	5.5
Respiratory diseases	830	5.2
Cancer	1014	6.4
Self-rated health	1952	12.2

Note:
Missing data were assumed to be completely random, and participants with missing information were excluded from the main analyses. Nevertheless, we also conducted multiple imputation as a sensitivity analysis.
