# Supplementary material for: Associations between cognitive activities and all-cause mortality among older adults with cognitive impairment: A prospective cohort study
Source: PLoS One. 2025 Feb 20;20(2):e0319093. doi: 10.1371/journal.pone.0319093 (PMC11841911; doi:10.1371/journal.pone.0319093)
Supplement: S3 Table — (PDF) [file pone.0319093.s003.pdf]

S3 Table. Baseline characteristics by reading books/newspapers

	Never	Sometimes	Almost everyday	p for trend
No. of participants	9854	341	252	
Sex: male	2485 (25.2%)	236 (69.2%)	195 (77.4%)	<0.001
Age (years)	95.0 (89.0–100.0)	91.0 (83.0–98.0)	91.0 (84.0–98.0)	<0.001
Education				<0.001
No school	8469 (85.9%)	77 (22.6%)	46 (18.3%)	
1 year or more	1385 (14.1%)	264 (77.4%)	206 (81.7%)	
Marital status				<0.001
Not in marriage	8661 (87.9%)	249 (73.0%)	175 (69.4%)	
In marriage	1193 (12.1%)	92 (27.0%)	77 (30.6%)	
Residence				<0.001
Urban	3335 (33.8%)	198 (58.1%)	165 (65.5%)	
Rural	6519 (66.2%)	143 (41.9%)	87 (34.5%)	
Co-residence				0.089
With family members	8198 (83.2%)	290 (85.0%)	223 (88.5%)	
Alone	1296 (13.2%)	34 (10.0%)	21 (8.3%)	
In an institution	360 (3.7%)	17 (5.0%)	8 (3.2%)	
Regular intake of fruits	2197 (22.3%)	103 (30.2%)	115 (45.6%)	<0.001
Regular intake of vegetables	7694 (78.1%)	272 (79.8%)	213 (84.5%)	0.014
Regular intake of meats	3545 (36.0%)	144 (42.2%)	138 (54.8%)	<0.001
Current smoking	1240 (12.6%)	78 (22.9%)	51 (20.2%)	<0.001
Current drinking	1746 (17.7%)	81 (23.8%)	66 (26.2%)	<0.001
Current regular exercise	1751 (17.8%)	116 (34.0%)	113 (44.8%)	<0.001
Hypertension	1357 (13.8%)	63 (18.5%)	39 (15.5%)	0.064
Diabetes	93 (0.9%)	10 (2.9%)	7 (2.8%)	<0.001
Heart diseases	589 (6.0%)	30 (8.8%)	31 (12.3%)	<0.001
Cerebrovascular diseases	354 (3.6%)	19 (5.6%)	20 (7.9%)	<0.001
Respiratory diseases	1067 (10.8%)	47 (13.8%)	33 (13.1%)	0.071
Cancer	26 (0.3%)	3 (0.9%)	1 (0.4%)	0.179
Self-rated health				<0.001
Poor	1608 (16.3%)	38 (11.1%)	32 (12.7%)	
Fair	3584 (36.4%)	121 (35.5%)	69 (27.4%)	
Good	4662 (47.3%)	182 (53.4%)	151 (59.9%)	
Playing cards/mah-jong				<0.001
Never	9303 (94.4%)	265 (77.7%)	212 (84.1%)	
Sometimes	395 (4.0%)	59 (17.3%)	24 (9.5%)	
Almost everyday	156 (1.6%)	17 (5.0%)	16 (6.3%)	
Watching TV or listening to radio				<0.001
Never	5394 (54.7%)	53 (15.5%)	34 (13.5%)	
Sometimes	2461 (25.0%)	141 (41.3%)	51 (20.2%)	
Almost everyday	1999 (20.3%)	147 (43.1%)	167 (66.3%)	
MMSE score	19.0 (13.0–22.0)	22.0 (18.0–23.0)	22.0 (17.8–23.0)	<0.001

Note:
Values are median (IQR) or n (%).
Abbreviations: IQR=interquartile range, MMSE=mini-mental state examination.
