# Supplementary material for: Associations between cognitive activities and all-cause mortality among older adults with cognitive impairment: A prospective cohort study
Source: PLoS One. 2025 Feb 20;20(2):e0319093. doi: 10.1371/journal.pone.0319093 (PMC11841911; doi:10.1371/journal.pone.0319093)
Supplement: S4 Table — (PDF) [file pone.0319093.s004.pdf]

S4 Table. Baseline characteristics by playing cards/mah-jong

	Never	Sometimes	Almost everyday	p for trend
No. of participants	9780	478	189	
Sex: male	2634 (26.9%)	210 (43.9%)	72 (38.1%)	<0.001
Age (years)	95.0 (89.0–100.0)	91.0 (83.0–99.0)	90.0 (84.0–97.0)	<0.001
Education				<0.001
No school	8136 (83.2%)	322 (67.4%)	134 (70.9%)	
1 year or more	1644 (16.8%)	156 (32.6%)	55 (29.1%)	
Marital status				<0.001
Not in marriage	8544 (87.4%)	389 (81.4%)	152 (80.4%)	
In marriage	1236 (12.6%)	89 (18.6%)	37 (19.6%)	
Residence				<0.001
Urban	3393 (34.7%)	216 (45.2%)	89 (47.1%)	
Rural	6387 (65.3%)	262 (54.8%)	100 (52.9%)	
Co-residence				<0.001
With family members	8184 (83.7%)	383 (80.1%)	144 (76.2%)	
Alone	1262 (12.9%)	59 (12.3%)	30 (15.9%)	
In an institution	334 (3.4%)	36 (7.5%)	15 (7.9%)	
Regular intake of fruits	2191 (22.4%)	153 (32.0%)	71 (37.6%)	<0.001
Regular intake of vegetables	7627 (78.0%)	391 (81.8%)	161 (85.2%)	0.002
Regular intake of meats	3564 (36.4%)	179 (37.4%)	84 (44.4%)	0.042
Current smoking	1212 (12.4%)	122 (25.5%)	35 (18.5%)	<0.001
Current drinking	1749 (17.9%)	105 (22.0%)	39 (20.6%)	0.034
Current regular exercise	1748 (17.9%)	146 (30.5%)	86 (45.5%)	<0.001
Hypertension	1363 (13.9%)	66 (13.8%)	30 (15.9%)	0.585
Diabetes	96 (1.0%)	10 (2.1%)	4 (2.1%)	0.010
Heart diseases	606 (6.2%)	29 (6.1%)	15 (7.9%)	0.486
Cerebrovascular diseases	369 (3.8%)	15 (3.1%)	9 (4.8%)	0.902
Respiratory diseases	1058 (10.8%)	58 (12.1%)	31 (16.4%)	0.015
Cancer	28 (0.3%)	2 (0.4%)	0 (0.0%)	0.802
Self-rated health				<0.001
Poor	1602 (16.4%)	57 (11.9%)	19 (10.1%)	
Fair	3569 (36.5%)	156 (32.6%)	49 (25.9%)	
Good	4609 (47.1%)	265 (55.4%)	121 (64.0%)	
Playing cards/mah-jong				<0.001
Never	9303 (95.1%)	395 (82.6%)	156 (82.5%)	
Sometimes	265 (2.7%)	59 (12.3%)	17 (9.0%)	
Almost everyday	212 (2.2%)	24 (5.0%)	16 (8.5%)	
Watching TV or listening to radio				<0.001
Never	5341 (54.6%)	101 (21.1%)	39 (20.6%)	
Sometimes	2443 (25.0%)	171 (35.8%)	39 (20.6%)	
Almost everyday	1996 (20.4%)	206 (43.1%)	111 (58.7%)	
MMSE score	19.0 (13.0–22.0)	21.0 (18.0–23.0)	21.0 (18.0–23.0)	<0.001

Note:

Values are median (IQR) or n (%).

Abbreviations: IQR=interquartile range, MMSE=mini-mental state examination.
