# Supplementary material for: Associations between cognitive activities and all-cause mortality among older adults with cognitive impairment: A prospective cohort study
Source: PLoS One. 2025 Feb 20;20(2):e0319093. doi: 10.1371/journal.pone.0319093 (PMC11841911; doi:10.1371/journal.pone.0319093)
Supplement: S5 Table — (PDF) [file pone.0319093.s005.pdf]

S5 Table. Baseline characteristics by watching TV or listening to radio

	Never	Sometimes	Almost everyday	p for trend
No. of participants	5481	2653	2313	
Sex: male	1287 (23.5%)	786 (29.6%)	843 (36.4%)	<0.001
Age (years)	97.0 (90.0–101.0)	93.0 (87.0–100.0)	91.0 (84.0–100.0)	<0.001
Education				<0.001
No school	4773 (87.1%)	2140 (80.7%)	1679 (72.6%)	
1 year or more	708 (12.9%)	513 (19.3%)	634 (27.4%)	
Marital status				<0.001
Not in marriage	4997 (91.2%)	2287 (86.2%)	1801 (77.9%)	
In marriage	484 (8.8%)	366 (13.8%)	512 (22.1%)	
Residence				<0.001
Urban	1546 (28.2%)	995 (37.5%)	1157 (50.0%)	
Rural	3935 (71.8%)	1658 (62.5%)	1156 (50.0%)	
Co-residence				0.005
With family members	4485 (81.8%)	2241 (84.5%)	1985 (85.8%)	
Alone	821 (15.0%)	290 (10.9%)	240 (10.4%)	
In an institution	175 (3.2%)	122 (4.6%)	88 (3.8%)	
Regular intake of fruits	970 (17.7%)	594 (22.4%)	851 (36.8%)	<0.001
Regular intake of vegetables	4081 (74.5%)	2123 (80.0%)	1975 (85.4%)	<0.001
Regular intake of meats	1733 (31.6%)	919 (34.6%)	1175 (50.8%)	<0.001
Current smoking	583 (10.6%)	366 (13.8%)	420 (18.2%)	<0.001
Current drinking	926 (16.9%)	487 (18.4%)	480 (20.8%)	<0.001
Current regular exercise	670 (12.2%)	585 (22.1%)	725 (31.3%)	<0.001
Hypertension	695 (12.7%)	382 (14.4%)	382 (16.5%)	<0.001
Diabetes	51 (0.9%)	21 (0.8%)	38 (1.6%)	0.016
Heart diseases	274 (5.0%)	176 (6.6%)	200 (8.6%)	<0.001
Cerebrovascular diseases	188 (3.4%)	85 (3.2%)	120 (5.2%)	0.001
Respiratory diseases	577 (10.5%)	291 (11.0%)	279 (12.1%)	0.054
Cancer	16 (0.3%)	4 (0.2%)	10 (0.4%)	0.484
Self-rated health				<0.001
Poor	1008 (18.4%)	357 (13.5%)	313 (13.5%)	
Fair	2076 (37.9%)	965 (36.4%)	733 (31.7%)	
Good	2397 (43.7%)	1331 (50.2%)	1267 (54.8%)	
Playing cards/mah-jong				<0.001
Never	5394 (98.4%)	2461 (92.8%)	1999 (86.4%)	
Sometimes	53 (1.0%)	141 (5.3%)	147 (6.4%)	
Almost everyday	34 (0.6%)	51 (1.9%)	167 (7.2%)	
Watching TV or listening to radio				<0.001
Never	5341 (97.4%)	2443 (92.1%)	1996 (86.3%)	
Sometimes	101 (1.8%)	171 (6.4%)	206 (8.9%)	
Almost everyday	39 (0.7%)	39 (1.5%)	111 (4.8%)	
MMSE score	17.0 (11.0–21.0)	20.0 (15.0–23.0)	21.0 (17.0–23.0)	<0.001

Note:
Values are median (IQR) or n (%).
Abbreviations: IQR=interquartile range, MMSE=mini-mental state examination.
