# Supplementary material for: Associations between cognitive activities and all-cause mortality among older adults with cognitive impairment: A prospective cohort study
Source: PLoS One. 2025 Feb 20;20(2):e0319093. doi: 10.1371/journal.pone.0319093 (PMC11841911; doi:10.1371/journal.pone.0319093)
Supplement: S6 Table — (PDF) [file pone.0319093.s006.pdf]

S6 Table. Associations of individual cognitive activities with all-cause mortality

	Never	Sometimes	Almost everyday
Reading books/newspapers			
Number of participants	9854	341	252
Number of deaths	8320	262	181
Total person-years of follow-up	31533.1	1179.9	919.1
Mortality rate (95% CI), per 1000 person-years	263.8 (259.0–268.7)	222.1 (198.3–245.8)	196.9 (171.2–222.7)
Unadjusted HR (95% CI), p	1.00 (ref)	0.84 (0.74–0.95), 0.005	0.74 (0.64–0.86), <0.001
Adjusted HR (95% CI), p			
model 1[a]	1.00 (ref)	0.92 (0.82–1.05), 0.220	0.77 (0.67–0.90), 0.001
model 2[b]	1.00 (ref)	0.89 (0.78–1.01), 0.065	0.75 (0.64–0.88), <0.001
Playing cards/mah-jong			
Number of participants	9780	478	189
Number of deaths	8268	354	141
Total person-years of follow-up	31115.8	1751.6	764.8
Mortality rate (95% CI), per 1000 person-years	265.7 (260.8–270.6)	202.1 (183.3–220.9)	184.4 (156.9–211.9)
Unadjusted HR (95% CI), p	1.00 (ref)	0.75 (0.68–0.84), <0.001	0.69 (0.58–0.81), <0.001
Adjusted HR (95% CI), p			
model 1[a]	1.00 (ref)	0.88 (0.79–0.98), 0.024	0.80 (0.68–0.95), 0.009
model 2[b]	1.00 (ref)	0.89 (0.80–0.99), 0.032	0.85 (0.72–1.00), 0.051
Watching TV or listening to radio			
Number of participants	5481	2653	2313
Number of deaths	4845	2160	1758
Total person-years of follow-up	15578.0	8961.7	9092.3
Mortality rate (95% CI), per 1000 person-years	311.0 (303.7–318.3)	241.0 (232.2–249.9)	193.3 (185.2–201.5)
Unadjusted HR (95% CI), p	1.00 (ref)	0.77 (0.73–0.81), <0.001	0.61 (0.58–0.65), <0.001
Adjusted HR (95% CI), p			
model 1[a]	1.00 (ref)	0.89 (0.84–0.93), <0.001	0.75 (0.70–0.79), <0.001
model 2[b]	1.00 (ref)	0.89 (0.84–0.93), <0.001	0.75 (0.71–0.79), <0.001

Note:

[a] Adjustment for sex and age.

[b] Adjustment for sex, age, education, marital status, residence, co-residence, regular intake of fruits, regular intake of vegetables, regular intake of meats, current smoking, current drinking, current regular exercise, hypertension, diabetes, heart diseases, cerebrovascular diseases, respiratory diseases, cancer, and self-rated health.

Abbreviations: CI=confidence interval, HR=hazard ratio.
