# Supplementary material for: Associations between cognitive activities and all-cause mortality among older adults with cognitive impairment: A prospective cohort study
Source: PLoS One. 2025 Feb 20;20(2):e0319093. doi: 10.1371/journal.pone.0319093 (PMC11841911; doi:10.1371/journal.pone.0319093)
Supplement: S7 Table — (PDF) [file pone.0319093.s007.pdf]

S7 Table. Mediation analysis of individual cognitive activities by baseline cognitive function

	Never	Sometimes	Almost everyday
Exposures: reading books/newspapers			
Mediators: baseline cognitive function (i.e., MMSE socre)			
Natural direct effect; HR (95% CI), p		0.90 (0.80–1.02), 0.098	0.75 (0.64–0.88), <0.001
Natural indirect effect; HR (95% CI), p		0.98 (0.97–1.00), 0.006	0.99 (0.98–1.00), 0.184
Mediation proportion; % (95% CI), p		13.0 (-26.8 to 87.2), 0.064	3.0 (-1.4 to 10.5), 0.184
Exposures: playing cards/mah-jong			
Mediators: baseline cognitive function (i.e., MMSE socre)			
Natural direct effect; HR (95% CI), p		0.92 (0.83–1.02), 0.114	0.86 (0.73–1.01), 0.056
Natural indirect effect; HR (95% CI), p		0.97 (0.96–0.98), <0.001	0.97 (0.96–0.99), <0.001
Mediation proportion; % (95% CI), p		27.9 (11.6 to 140.2), 0.024	14.7 (4.5 to 65.9), 0.026
Exposures: watching TV or listening to radio			
Mediators: baseline cognitive function (i.e., MMSE socre)			
Natural direct effect; HR (95% CI), p		0.91 (0.86–0.96), 0.002	0.77 (0.73–0.82), <0.001
Natural indirect effect; HR (95% CI), p		0.97 (0.96–0.98), <0.001	0.97 (0.96–0.98), <0.001
Mediation proportion; % (95% CI), p		24.2 (15.0 to 44.7), <0.001	9.4 (6.7 to 13.2), <0.001

Note:
Natural direct effect and natural indirect effect estimated the effect of individual cognitive activities on all-cause mortality that did not or did act through the mediator (i.e., baseline cognitive function), respectively. The mediation proportion estimated the percentage of the effect of individual cognitive activities, on the log(HR) scale, that acted through the mediator (i.e., baseline cognitive function). The model was adjusted for sex, age, education, marital status, residence, co-residence, regular intake of fruits, regular intake of vegetables, regular intake of meats, current smoking, current drinking, current regular exercise, hypertension, diabetes, heart diseases, cerebrovascular diseases, respiratory diseases, cancer, and self-rated health.
Abbreviations: CI=confidence interval, HR=hazard ratio, MMSE=mini-mental state examination.
