# Supplementary material for: Associations between cognitive activities and all-cause mortality among older adults with cognitive impairment: A prospective cohort study
Source: PLoS One. 2025 Feb 20;20(2):e0319093. doi: 10.1371/journal.pone.0319093 (PMC11841911; doi:10.1371/journal.pone.0319093)
Supplement: S8 Table — (PDF) [file pone.0319093.s008.pdf]

S8 Table. Mediation analysis of baseline cognitive function by cognitive activities

	HR (95% CI), p; % (95% CI), p
Exposures: baseline cognitive function (i.e., MMSE socre)	
Mediators: no. of cognitive activities	
Total effect	0.980 (0.976–0.985), <0.001
Natural direct effect	0.982 (0.979–0.986), <0.001
Natural indirect effect	0.998 (0.996–1.00), 0.086
Mediation proportion	11.4 (-1.0 to 18.9), 0.086
Exposures: baseline cognitive function (i.e., MMSE socre)	
Mediators: reading books/newspapers	
Total effect	0.981 (0.977–0.984), <0.001
Natural direct effect	0.980 (0.977–0.984), <0.001
Natural indirect effect	1.000 (0.999–1.001), 0.738
Mediation proportion	-0.8 (-4.6 to 6.0), 0.738
Exposures: baseline cognitive function (i.e., MMSE socre)	
Mediators: playing cards/mah-jong	
Total effect	0.981 (0.977–0.984), <0.001
Natural direct effect	0.981 (0.977–0.984), <0.001
Natural indirect effect	1.000 (0.999–1.000), 0.630
Mediation proportion	0.0 (-1.8 to 2.8), 0.630
Exposures: baseline cognitive function (i.e., MMSE socre)	
Mediators: watching TV or listening to radio	
Total effect	0.981 (0.976–0.985), <0.001
Natural direct effect	0.982 (0.978–0.986), <0.001
Natural indirect effect	0.999 (0.996–1.001), 0.186
Mediation proportion	5.9 (-3.4 to 16.6), 0.186

Note:

For this section of the statistical analysis, we performed a regression-based causal mediation analysis within the direct counterfactual framework to evaluate how cognitive activities mediate the association between baseline cognitive function (i.e., MMSE socre) and all-cause mortality. In brief, two regression models were fitted: one for the outcome (i.e., all-cause mortality; using Cox models) and another for the mediator (i.e., cognitive activities; using ordinal logistic regression models). In the two models, baseline cognitive function (i.e., MMSE socre) was considered as a continuous variable, and HR was given per 1-point of MMSE socre; while cognitive activities served as ordinal categorical variables. Standard errors were estimated via bootstrapping with 1000 samples. All models were adjusted for potential confounders, including sex, age, education, marital status, residence, co-residence, regular intake of fruits, regular intake of vegetables, regular intake of meats, current smoking, current drinking, current regular exercise, hypertension, diabetes, heart diseases, cerebrovascular diseases, respiratory diseases, cancer, and self-rated health.

For the result, natural direct effect and natural indirect effect estimated the effect of baseline cognitive function (i.e., MMSE socre) on all-cause mortality that did not or did act through the mediator (i.e., cognitive activities), respectively. The mediation proportion estimated the percentage of the effect of baseline cognitive function (i.e., MMSE socre), on the log(HR) scale, that acted through the mediator (i.e., cognitive activities).

Abbreviations: CI=confidence interval, HR=hazard ratio, MMSE=mini-mental state examination.
