# Supplementary figures and images for: Associations between cognitive activities and all-cause mortality among older adults with cognitive impairment: A prospective cohort study
Source: PLoS One. 2025 Feb 20;20(2):e0319093. doi: 10.1371/journal.pone.0319093 (PMC11841911; doi:10.1371/journal.pone.0319093)

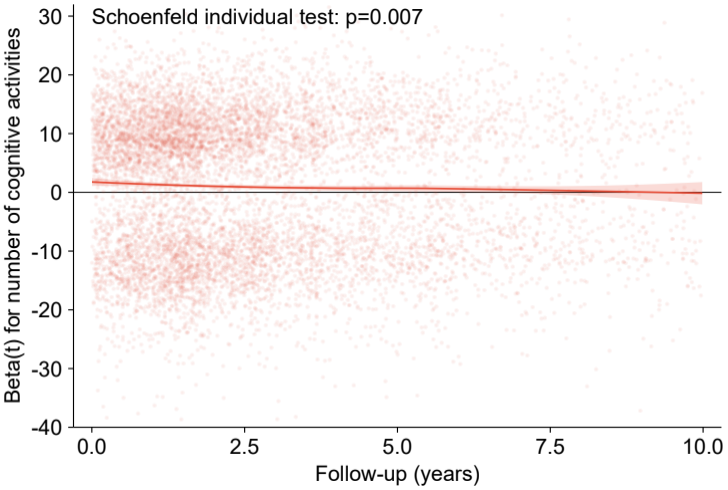

Supplement: S1 Fig — Note: The proportional-hazards assumption is an important premise for Cox proportional-hazards models; however, no hazards are perfectly proportional in nearly any clinical study (JAMA 2020;323:1401–1402). In small to moderate-sized samples, statistical tests may fail to reject such model assumptions due to insufficient power; however, even minor violations of the assumption can become evident with a sufficiently large sample size. Indeed, even when the model only approximately satisfies the proportional-hazards assumption, it can still yield reasonably accurate inferences (Am J Epidemiol 2024;193:926–927). With this consideration in mind, it’s best to use a combination of statistical tests and visual tests to determine the most serious violations. For statistical tests, we assessed the proportional-hazards assumption using the `cox.zph` function from the `survival` package, applying a p-value threshold of 0.001 (①Am J Epidemiol 2024;193(6):926–927; ②Nat Med 2024;30:85–97). For visual tests, we visually assessed this assumption by examining the relationship between β(t) of exposures and the timescale. We found no evidence indicating a potential violation of the proportional-hazards assumption for the exposure-outcome association, with a p-value from the Schoenfeld individual tests exceeding 0.001. In addition, the relationship between β(t) of exposures and the timescale could be characterized as approximately horizontal and constant. Overall, both statistical analyses and graphical representations supported that the proportional-hazards assumption was satisfied, or at least approximately met, allowing for data analysis using Cox proportional-hazards models. (PDF) [file pone.0319093.s009.pdf]

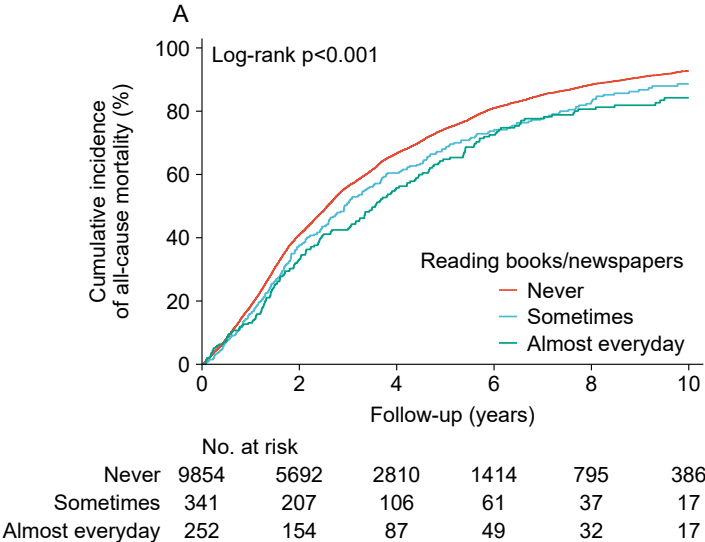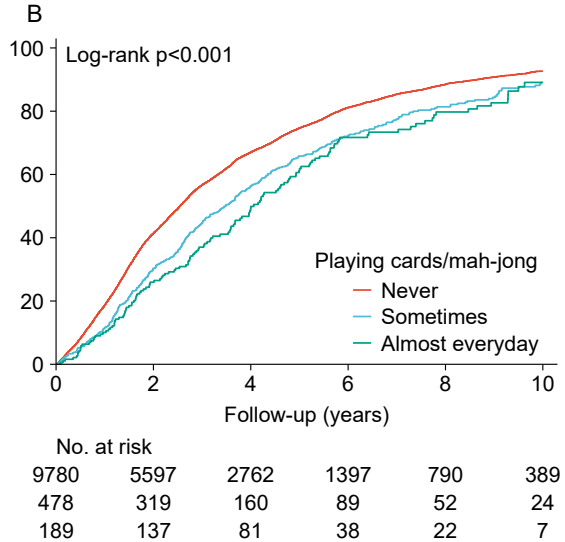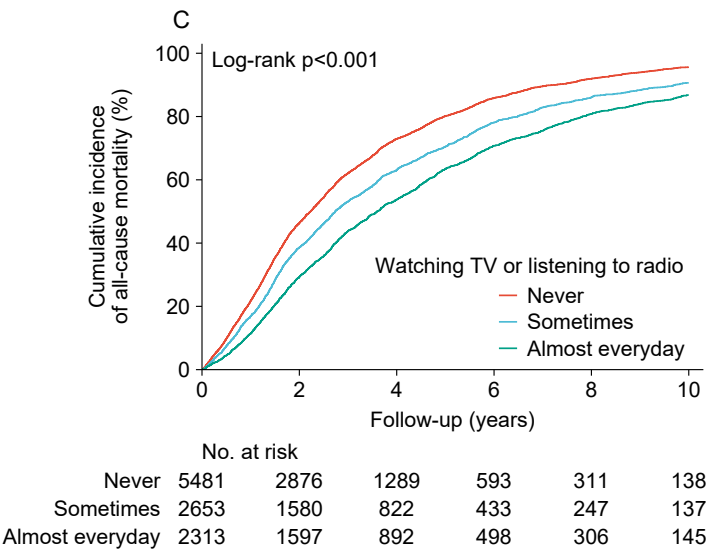

Supplement: S2 Fig — (PDF) [file pone.0319093.s010.pdf]

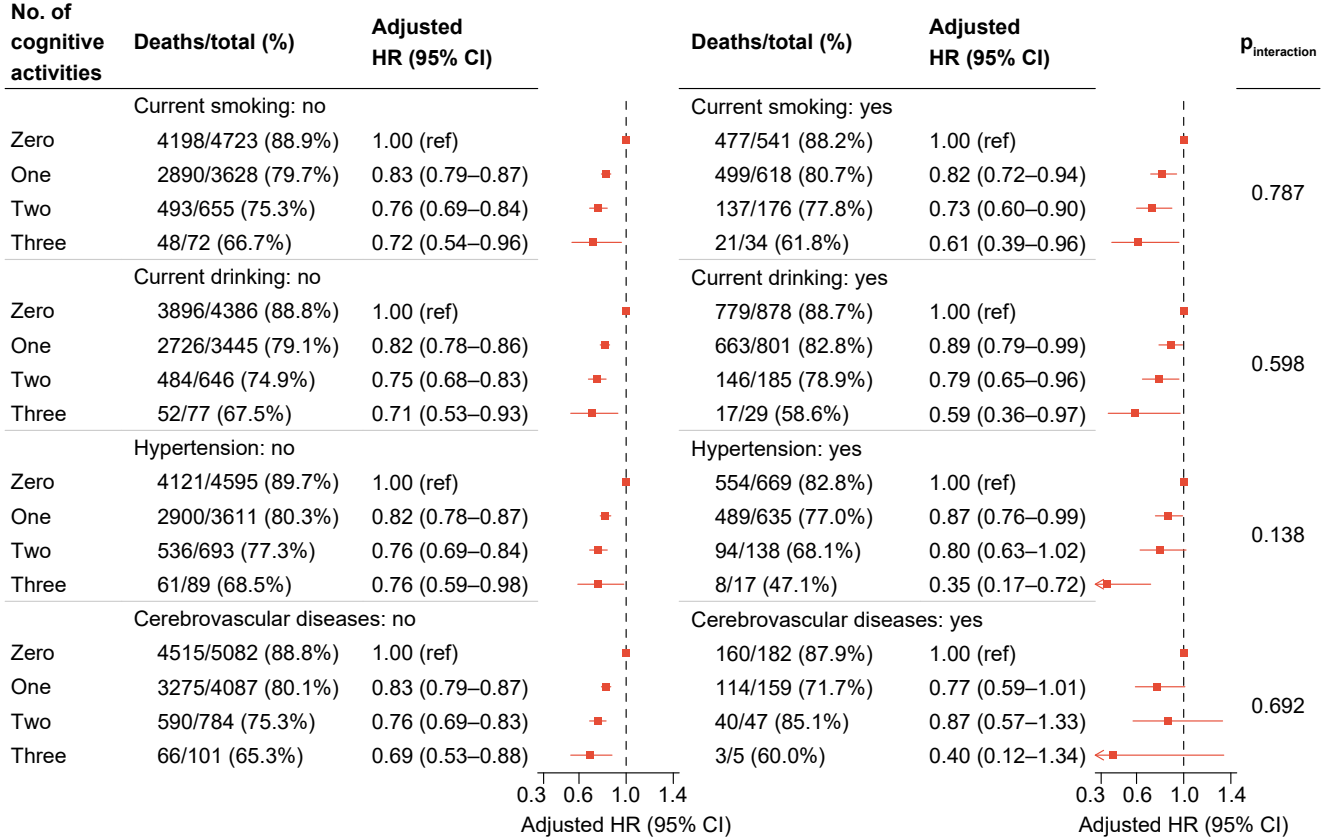

Supplement: S3 Fig — Note: Each stratification with adjustment for all factors (sex, age, education, marital status, residence, co-residence, regular intake of fruits, regular intake of vegetables, regular intake of meats, current smoking, current drinking, current regular exercise, hypertension, diabetes, heart diseases, cerebrovascular diseases, respiratory diseases, cancer, and self-rated health) except the stratification factor itself. (PDF) [file pone.0319093.s011.pdf]
